# Radiographic and Tomographic Study of the Cranial Bones in Children with the Idiopathic Type of West Syndrome

**DOI:** 10.3390/pediatric16020035

**Published:** 2024-05-24

**Authors:** Ali Al Kaissi, Sergey Ryabykh, Farid Ben Chehida, Hamza Al Kaissi, Vasileios Dougales, Vladimir M. Kenis, Franz Grill

**Affiliations:** 1 National Medical Research Center for Traumatology and Orthopedics n.a. G.A. Ilizarov, 640014 Kurgan, Russia; 2 Veltischev Clinical Institute, Pirogov Russian National Research Medical University, 117997 Moscow, Russia; rso_@mail.ru; 3 Ibn Zohr Institute of Diagnostic Radiology, Tunis 1004, Tunisia; if.chehida@gnet.tn; 4 IhreHaut Dermatology Center, 87600 Kaufbeuren, Bavaria, Germany; alkaissihamza@gmail.com; 5 Department of Orthopedic Surgery and Traumatology, Kantonsspital Aarau, 130021 Arau, Switzerland; vasileios.dougalis@gmail.com; 6 Department of Foot and Ankle Surgery, Neuroorthopaedics and Systemic Disorders, Pediatric Orthopedic Institute, Parkovaya Str., 64-68, Pushkin, 196605 Saint Petersburg, Russia; kenis@mail.ru; 7 Pediatric Department, Orthopedic Hospital of Speising, 1130 Vienna, Austria; grill.franzleo@gmail.com

**Keywords:** idiopathic type of West syndrome, cryptogenic epileptic spasms, cranial bones, radiology, CT scan

## Abstract

Background: Neither radiological phenotypic characteristics nor reconstruction CT scan has been used to study the early anatomical disruption of the cranial bone in children with the so-called idiopathic type of West syndrome. Material and Methods: The basic diagnostic measures and the classical antiepileptic treatments were applied to these children in accordance with the conventional protocol of investigations and treatment for children with West syndrome. Boys from three unrelated families were given the diagnosis of the idiopathic type of West syndrome, aged 7, 10 and 12 years old. Parents underwent extensive clinical examinations. Three parents (age range of 28–41 year) were included in this study. All children showed a history of intellectual disabilities, cryptogenic epileptic spasms and fragmented hypsarrhythmia. These children and their parents were referred to our orthopedic departments because of variable skeletal deformities. Variable forms of skeletal deformities were the motive for the families to seek orthopedic advice. A constellation of flat foot, torticollis and early-onset osteoarthritis were observed by the family doctor. Apparently, and from the first clinical session in our practice, we felt that all these children are manifesting variable forms of abnormal craniofacial contour. Thereby, we immediately performed detailed cranial radiological phenotypic characterization of every affected child, as well as the siblings and parents, and all were enrolled in this study. All affected children underwent whole-exome sequence analysis. Results: The craniofacial phenotype of all children revealed apparent developmental anatomical disruption of the cranial bones. Palpation of the skull bones showed unusual palpable bony ridges along different sutural locations. A 7-year-old child showed abnormal bulging over the sagittal suture, associated with bilateral bony ridges over the squamosal sutures. AP skull radiograph of a 7-year-old boy with West syndrome showed facial asymmetry with early closure of the metopic suture, and other sutures seemed ill-defined. A 3D reconstruction CT scan of the skull showed early closure of the metopic suture. Another 3D reconstruction CT scan of the skull while the patient was in flexion showed early closure of the squamosal sutures, pressing the brain contents upward, causing the development of a prominent bulge at the top of the mid-sagittal suture. A reformatted 3D reconstruction CT scan confirmed the bilateral closure of the squamosal suture. Examination of the parents revealed a similar skull radiographic abnormality in his mother. A 3D reformatted frontal cranial CT of a 35-year-old mother showed early closure of the metopic and sagittal sutures, causing a mid-sagittal bony bulge. A 10-year-old boy showed an extremely narrow frontal area, facial asymmetry and a well palpable ridge over the lambdoid sutures. A 3D axial reconstruction CT scan of a 10-year-old boy with West syndrome illustrated the asymmetry of the posterior cranial bones along the lambdoid sutures. Interestingly, his 28-year-old mother has been a client at the department of spine surgery since she was 14 years old. A 3D reconstruction CT scan of the mother showed a noticeable bony ridge extending from the metopic suture upwards to involve the sagittal suture (red arrow heads). The black arrow shows a well demarcated bony ridge over the squamosal suture. A 3D reconstruction CT scan of the skull and spine showed the thick bony ridge of the metopic and the anterior sagittal as well as bilateral involvement of the squamosal, causing apparent anterior narrowing of the craniofacial contour. Note the lumbar scoliosis. A 12-year-old boy showed brachycephaly. A lateral skull radiograph of a 12-year-old boy with West syndrome showed premature sutural fusion, begetting an abnormal growth pattern, resulting in cranial deformity. The nature of the deformity depends on which sutures are involved, the time of onset and the sequence in which individual sutures fuse. In this child, brachycephalic secondary to craniosynostosis, which occurred because of bilateral early ossification of the coronal sutures, led to bi-coronal craniosynostosis. Thickened frontal bones and an ossified interclinoid ligament of the sella turcica were encountered. The lateral skull radiograph of a 38-year-old mother with a history of poor schooling achievements showed a very similar cranial contour of brachycephaly, thickening of the frontal bones and massive ossification of the clinoid ligament of the sella turcica. Maternal history revealed a history of multiple spontaneous miscarriages in the first trimester of more than five times. Investigating his parents revealed a brachycephalic mother with borderline intelligence. We affirm that the pattern of inheritance in the three boys was compatible with the X-linked recessive pattern of inheritance. Whole-exome sequencing showed non-definite phenotype/genotype correlation. Conclusions: The aim of this study was sixfold: firstly, to refute the common usage of the term idiopathic; secondly, we feel that it could be possible that West syndrome is a symptom complex rather than a separate diagnostic entity; thirdly, to further detect the genetic carrier, we explored the connection between the cranial bones in children with West syndrome with what has been clinically observed in their parents; fourthly, the early life anatomical disruptions of the cranial bones among these children seem to be heterogeneous; fifthly, it shows that the progressive deceleration in the development of this group of children is highly connected to the progressive closure of the cranial sutures; sixthly, we affirm that our findings are novel.

## 1. Introduction

West syndrome is characterized by early epileptic seizures in infancy or possibly occurring later on in early childhood (cryptogenic epileptic spasms). West syndrome is an unpleasant type of childhood epilepsy because of uncontrolled seizures, which are associated with different grades of intellectual disability [1,2].

The etiological understanding of West syndrome encompasses several pathological/syndromic entities, in which West syndrome is a symptom complex rather than a definite diagnostic entity. Aicardi syndrome is an X-linked dominant condition, characterized by infantile spasms and chorio-retinopathy, which is almost distinctive in having footprint-shaped lacunae and agenesis of the corpus callosum. Additional malformations include staphyloma, coloboma of the optic nerve, microphthalmia and vertebral body anomalies, such as hemivertebrae, vertebral malsegmentation, scoliosis and abnormal costovertebral articulations. Some patients manifested cleft lip and palate [3,4,5,6]. Schinzel–Giedion syndrome (SGS) is characterized by seizures and abnormal EEG as well as global developmental delays and intellectual disability. SGS is characterized by distinctive clinical features, specifically abnormal craniofacial contour, as viewed from the front the forehead, which is tall and prominent, and there is severe temporal narrowing, leading to mid-face retraction. Hypoplasia/agenesis of the corpus callosum, hydrocephaly and large ventricles are the main cerebral malformation complex [7,8,9].

In sotos syndrome (cerebral gigantism), infants are born with increased birthweight, with an enlarged head circumference. Infantile spasm occurs in connection with dilated cerebral ventricles and in some hypoplasia of the corpus callosum, with the common occurrence of cavum as well as variable cerebral abnormalities [10,11,12,13,14]. The classical type of Alexander’s disease is characterized by infantile onset with seizures, neuro-degeneration and spasticity. Important clinical signs include macrocephaly, hydrocephaly and large ventricles. In some cerebral atrophy/myelin abnormalities, most cases die within five years [15,16,17,18]. West syndrome can occur in children with variable forms of metabolic disorders. Pyruvate dehydrogenase complex deficiency is an example, which is a major etiology behind congenital lactic acidemia, in which seizures are a major clinical presentation [19,20]. West syndrome has also been described in the literature as Salaam tics or Blitz–Nick–Salaam Krämpfe as a form of generalized/difficult forms of seizures associated with characteristic EEG readings [21]. 

The eponym hypsarrhythmia was introduced by Gibbs and Gibbs to describe an EEG pattern commonly associated with West syndrome. The pattern consists of high-voltage arrhythmic slow waves mixed with spike discharges, showing a multifocal distribution. It should be noted that hypsarrhythmia refers only to the EEG pattern and should not be used to describe the clinical phenotype. In other words, it is important not to classify all large-amplitude EEGs as hypsarrhythmia, especially when activities are regular and rhythmic or when no discharges are seen. Hypsarrhythmia is accepted as an age-dependent EEG pattern, although it may occur in previously healthy children with co-existing abnormalities of variable etiological background. Hypsarrhythmia may develop as a result of any early cerebral insult, which is either genetically determined or of environmental etiology. The cerebral insult can be focal or multifocal, such as infarction, porencephalic cysts, vascular (microangiopathy) or in infants with developmental abnormalities, and may even develop in rare cases of young infants with supratentorial tumors [22].

It is well known that the most difficult aspect of any long-term disability is the proper understanding of the correlated anatomical disruption, which can enhance the explanation of the pathological series within the natural history of the disease. We wish to stress that, in our experience, the genotype can rarely interpret the extent of the skeletal malformation complex and/or the natural history of the disease. To alleviate the burden on these families and their children and to clarify the reason behind the frequent clinical complaints and hospitalizations, we referred to profound tomographic analysis of the cranial bones.

## 2. Materials and Methods

This study was carried out between 2016 and 2020, within the children orthopedic departments of Speising (Vienna, Austria), Ilizarov Institute of orthopedics (Kurgan, Russia), Ibn Zohr Institute of Diagnostic Radiology (Tunis, Tunisia) and the department of Foot and Ankle Surgery, Neuroorthopaedics and Systemic Disorders, Pediatric Orthopedic Institute (Saintpetersburg, Russia).

The study protocol was approved by the Ethics Committee of the Ilizarov Scientific Research Institute (No. 4(50)/13.12.2016, Kurgan, Russia). Informed consent was obtained from the patient’s guardians. We fully documented the affected children, siblings and parents by means of comprehensive clinical and radiological studies of each individual. The senior author with the assistance of colleagues in Vienna and abroad agreed upon a clear strategy of comprehensive clinical documentation. Three boys aged 7–10 and 12 years old from three unrelated families were diagnosed with the idiopathic type of West syndrome by different pediatric neurological institutes. Recently, these children were referred to our orthopedic departments because of a diverse form of skeletal deformities, such as flat foot, ligamentous hyperlaxity and early-onset osteoarthritis. Clinical examination of parents has been considered a cornerstone of the diagnostic process. Our major clinical strategy is primarily based on detailed clinical and radiological documentation. Family history and multigenerational exploration of family subjects are mandatory when dealing with patients with unknown etiology (idiopathic). We gave excessive attention to a maternal history of spontaneous miscarriages, stillbirths, weak or hyperactive in utero fetal movements, hyperemesis gravidarum, stillbirths, perinatal mortalities and a history of sudden infant death syndrome. Unusual gestational events are essential markers required for proper management. Clinical examination of parents can be of tremendous help to understand the mode of the transmitted gene. Meanwhile, assessing the educational levels of the parents was a priority. The history of these mothers showed borderline intelligence and a history of poor schooling achievements. The detailed clinical examination of these children was followed by a skeletal survey. All laboratory investigations were negative, and karyotyping of lymphocytes from peripheral blood with GTG banding was normal (46, XY). Full hematological investigations revealed nothing of significance. Syndromic craniosynostosis with FGFRs and TWIST gene mutation were ruled out. Full hematological investigations revealed nothing of significance. Whole-exome sequencing showed no definite reason. We think that it is highly likely that the applied technique missed or did not include the required part of the DNA that might be responsible for causing the constellation of disorders in this group of children. The first and foremost notable clinical observation was the variable abnormality in the craniofacial phenotype in all children. Interestingly, we detected similar cranial abnormalities in all mothers. All children underwent a series of conventional radiographs, tomographic studies and MRI imaging. Interestingly, despite the anatomical disruption in the development of the calvaria, cerebral MRI showed no concomitant pre-existing pathology.

## 3. Results

### 3.1. Family 1

**Figure 1:**
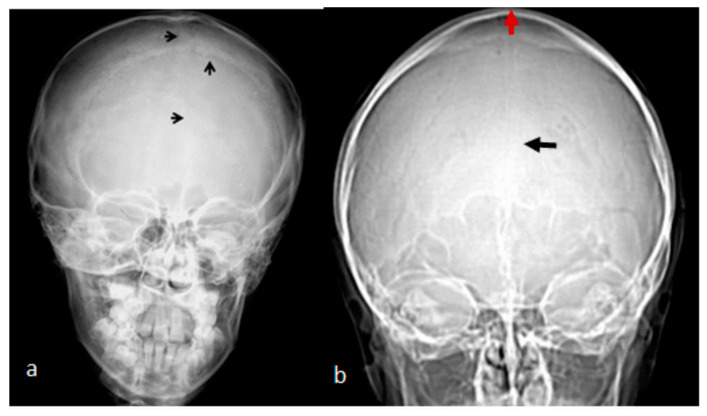
(**a**). AP skull radiograph of a 7-year-old boy with West syndrome showed facial asymmetry with early closure of the metopic suture (arrow head) similarly the coronal as well as the sagittal sutures showed post-closure sclerosis; (**b**) A 3D reformatted frontal cranial CT of a 35-year-old mother clearly showed the closure of the metopic (arrow) and the sagittal sutures causing a mid-sagittal bony bulge (red-arrow).

**Figure 2:**
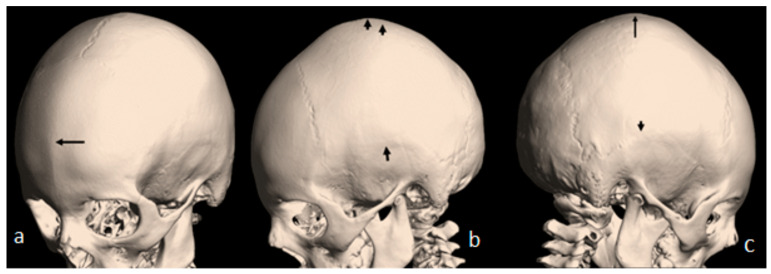
(**a**) A 3D reconstruction CT scan of the 7-year-old-child with mild flexion of the skull showed early closure of the metopic suture (arrow). (**b**) A 3D reconstruction CT scan showed early closure of the squamosal sutures (arrow), pressing the brain contents upward, causing the development of a prominent bulge at the top of the mid-sagittal sutures two arrows. (**c**) Another 3D reconstruction CT scan confirmed the bilateral closure of the squamosal suture (arrow head). The vertical arrow showed abnormal bulging of the vertex secondary to bilateral pressure exerted by the squamosal sutures.

A 7-year-old boy with West syndrome associated with hyperkinetic disorder was referred to our department because of ligamentous hyperlaxity and flat foot. Clinical examination showed growth deficiency (10th percentile). Surprisingly, he manifested an abnormal cranial phenotype almost typical for his mother. Palpation of his skull showed a bony ridge over the metopic suture, associated with an unusual bulge over the mid-sagittal suture. Bilateral bony ridges over the squamosal sutures were well delineated. Palpation of his 35-year-old-mother’s skull showed a prominent bony bulge over her sagittal suture. AP skull radiograph of a 7-year-old boy with West syndrome showed facial asymmetry with early closure of the metopic suture (arrow head) similarly the coronal as well as the sagittal sutures showed post-closure sclerosis (Figure 1a). A 3D reformatted frontal cranial CT of a 35-year-old mother clearly showed the closure of the metopic (arrow) and the sagittal sutures causing a mid-sagittal bony bulge (red-arrow) (Figure 1b). A 3D reconstruction CT scan of the 7-year-old-child with mild flexion of the skull showed early closure of the metopic suture (arrow) (a). A 3D reconstruction CT scan showed early closure of the squamosal sutures (arrow), pressing the brain contents upward, causing the development of a prominent bulge at the top of the mid-sagittal sutures two arrows (Figure 2b). Another 3D reconstruction CT scan confirmed the bilateral closure of the squamosal suture (arrow head). The vertical arrow showed abnormal bulging of the vertex secondary to bilateral pressure exerted by the squamosal sutures (c).

### 3.2. Family 2

**Figure 3:**
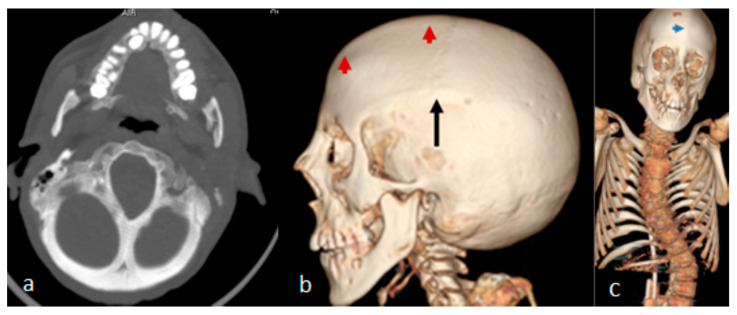
(**a**) A 3D axial reformatted CT scan of the 10-year-old boy illustrated the asymmetry of the posterior cranial bones along the lambdoid sutures; (**b**) The black arrow shows a well-demarcated boney ridge over the squamosal suture. (**c**) A 3D reconstruction CT scan of the skull and spine of the mother note the thoraco-lumbar scoliosis.

A 10-year-old boy received the diagnosis of West syndrome at the age of 3 and was referred to our department because of torticollis. Clinical examination showed a growth deficiency (10th percentile). Craniofacial asymmetry was a noticeable clinical feature. Examining the skull, we noticed asymmetrical massive bony ridges over the lambdoid sutures, with apparent but asymmetrical bulging of the occiput. The asymmetry was marked over the left portion of the left lambdoid, causing a bigger cranial compartment of the left over the right side of the occiput. A 3D axial reformatted CT scan of the 10-year-old boy illustrated the asymmetry of the posterior cranial bones along the lambdoid sutures Figure 3a). Interestingly, his 28-year-old mother has been a client at the department of spine surgery since she was 14. A 3D reconstruction CT scan of the mother showed a noticeable bony ridge extending from the metopic suture upwards to involve the sagittal suture (red arrow heads). The black arrow showed a well-demarcated boney ridge over the squamosal suture (Figure 3b). A 3D reconstruction CT scan of the skull and spine showed the thick bony ridge of the metopic and the anterior sagittal as well as bilateral involvement of the squamosal, causing apparent anterior narrowing of the craniofacial contour (blue arrow). Note the thoraco-lumbar scoliosis (Figure 3c).

### 3.3. Family 3

A 12-year-old boy with the diagnosis of West syndrome was referred to our department because of early-onset osteoarthritis. Interestingly, the child’s craniofacial contour resembles his 38-year-old mother.

**Figure 4:**
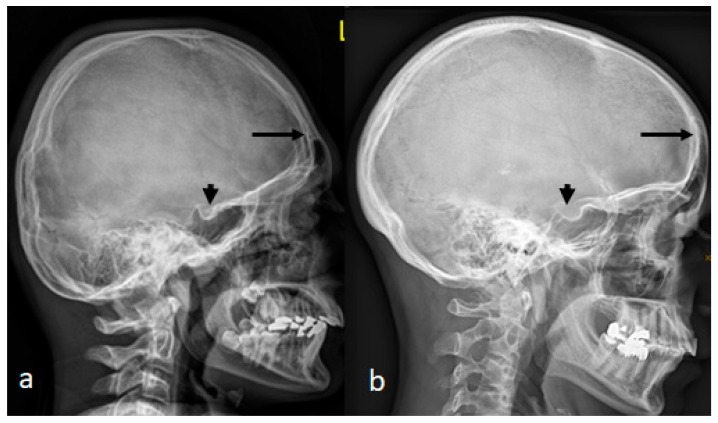
(**a**) AP skull radiograph of a-12-year-old boy showed brachycephalic skull secondary to craniosynostosis occurred because of bilateral early ossification of the coronal sutures, leading to bi-coronal craniosynostosis. Note the ossified interclinoid ligament of the sella turcica. (**b**) A lateral skull radiograph of a 38-year-old mother with a history of poor schooling achievements showed a very similar cranial contour of brachycephaly and massive ossification of the clinoid ligament of the sella turcica.

A lateral skull radiograph of a 12-year-old boy with West syndrome showed premature sutural fusion, begetting an abnormal growth pattern, resulting in cranial deformity (brachycephaly). The nature of the deformity depends on which sutures are involved, the time of onset and the sequence in which individual sutures fuse. In this child, brachycephalic secondary to craniosynostosis occurred because of bilateral early ossification of the coronal sutures, leading to bi-coronal craniosynostosis. Note the ossified interclinoid ligament of the sella turcica (Figure 4a). A lateral skull radiograph of a 38-year-old mother with a history of poor schooling achievements showed a very similar cranial contour of brachycephaly and massive ossification of the clinoid ligament of the sella turcica. Maternal history revealed a history of multiple spontaneous miscarriages in the first trimester of more than five times.

## 4. Discussion

West syndrome is a well-known sub-variety of infantile spasm syndrome and is considered the most common. 

The age of onset of West syndrome is variable (can occur between the early months of life to early childhood). West syndrome patients manifest a constellation of three main diagnostic elements of cryptogenic epileptic spasms, awkward deceleration of development/intellectual disability (though it can be within normal limits) as well as an interictal EEG pattern (fragmented hypsarrhythmia). The etiological understanding is a decisive and paramount basic tool from which the route of the natural history of the disease can be comprehended [23,24].

Previous studies described the genetic background of West syndrome as being vast and correlated with diverse forms of genetic background. 

Bruyere et al. performed a multi-generational family study in Canada; they observed the occurrence of West syndrome in boys, though the mothers showed no signs. They confirmed in their study that West syndrome is an X-linked recessively inherited disorder (mapped to Xp11.4-Xp22.11) [25].

Claes et al. studied the genetic marker in two families with West syndrome and described the disorder as X-linked West syndrome, mapped to Xp21.3-Xp22.1 [26].

Stromme et al. further described ARX gene mutation coding for an aristaless-related homeobox protein. A polyalanine expansion was encountered in the aforementioned families, as described by Bruyere et al. and Claes et al., and compared to a previous study by Stromme et al. [27,28].

Stromme and co-workers studied the clinical data from fifty intellectually disabled patients with the ARX mutation. They concluded that seizures were encountered in 29 patients; also, one family with a novel myoclonic epilepsy syndrome was associated with a missense mutation. Seventeen patients had infantile spasms. Other phenotypes were variable and ranged between mild to moderate intellectual disability. Some of the patients with intellectual disability showed dystonia, ataxia and also autism [29].

Scheffer et al. published a study on an Australian family manifesting the ARX gene mutation. The family presented with seizures and developmental delays. The findings in this family showed intellectual disabilities, and the carrier females had hyperreflexia. The authors named this condition X-linked recessive myoclonic epilepsy with spasticity and intellectual disability in boys XMESID [30].

Elia et al. described three boys with early-onset intractable epilepsy (drug-resistant myoclonic, tonic or infantile spasms), with profound intellectual disability and CDKL5 mutations [31].

Interestingly CDKL5 mutations were confirmed in a girl in and boy patients who showed features of Rett syndrome [32,33].

Striano et al. described two West syndrome patients with duplications of 14q12, including FOXG1, who showed no X-linked recessive trait [34].

Yuskaitis et al. assessed the clinical phenotype/genotypes of 131 patients with the idiopathic type of infantile spasms. Through retrospective analysis of the medical records, imaging and EEG results, they focused on the main two elements of delayed development and the associated seizures as the foremost key factors in patients with infantile spasms of unknown etiology [35].

None of the abovementioned studies described the cranial and skeletal development of individuals and parents with West syndrome, and most of the studies emphasized the genetic markers of the disease.

## 5. Conclusions

On the one hand, unusual craniofacial features are usually suspected in infants and children with well-known recognizable syndromes. Infants who present with less noticeable striking craniofacial features are almost ignored and rarely receive further concerns. On the other hand, pediatricians are almost always drawn to infants with unusual facies with a diagnosis of Down’s syndrome. Similarly, infants born with distorted skeletal development, regardless of the severity, are almost always diagnosed with achondroplasia, rushing for karyotyping in the former and genetic investigations for the latter. When the results showed negative results, the term idiopathic was immediately applied. The significance of the etiological understanding in children/adults with long-term ailments is to establish well-structured management. The clinical/radiological phenotypes are the main indices that guide the physician towards a definite and unbiased diagnosis. Our clinical strategy was directed towards an optimal level of etiological understanding as a top requirement to overcome any emotional or psychosomatic complications for both the children and their families. If any skeletal or extra-skeletal abnormality is present, it should be carefully studied and evaluated via comprehending its nature, severity, the degree of disability anticipated and the efficacy of treatment. In all types of disabilities, treatment is sometimes wrongly applied in connection with the symptoms (symptomatic treatment) and the actual cause left buried. Thereby, patients should be evaluated as a whole, from the point of their physical potential, dysmorphic features, whether minor or major, family history and examination of parents. All these markers can represent an important element to approach the actual diagnosis, rather than a superficial diagnosis. Disabilities of the musculoskeletal system encompass a broad spectrum of etiological backgrounds. The final objective in any diagnostic process is to approach what has been called the “Precise classification” of disabilities. The latter can never be achieved unless the clinician succeeds in connecting the disability to one of the etiological backgrounds: osteogenic, neurogenic, myogenic or stemming from other reasons. The conspicuous craniofacial features observed in three unrelated families with West syndrome highlight the necessity of a comprehensive clinical examination as a fundamental element in detecting the etiological understanding in patients with West syndrome. We wish to point out that the main limitation in our study is the small number of patients with a diagnosis of West syndrome. Nevertheless, we believe that the results of our findings can be used as an impactful inducement to further clarify the etiological understanding in children with the idiopathic type of West syndrome. We stress the necessity of correlating the clinical phenotype via precise conduct of high-quality clinical and investigative efforts. Despite the limited number of children and parents in this study, the abnormal cranial contour in children with West syndrome can be considered important for future assessment.

## Data Availability

The data presented in this study are available on request from the corresponding author.
